# 随访观察——主病灶切除的胸腔内播散腺癌或鳞癌患者的可选治疗策略

**DOI:** 10.3779/j.issn.1009-3419.2018.04.14

**Published:** 2018-04-20

**Authors:** 英 陈, 巍 李, 文芳 唐, 学宁 杨, 文昭 钟

**Affiliations:** 1 510515 广州，南方医科大学第二临床医学院 The Second Medical College of Southern Medical University, Guangzhou 510515, China; 2 510080 广州，广东省肺癌研究所，广东省人民医院 & 广东省医学科学院 Guangdong Provincial Institute of Lung Cancer, Guangdong Provincial People' s Hospital & Guangdong Academy of Medical Sciences, Guangzhou 510080, China; 3 518000 佛山，佛山市第一人民医院 Foshan First People Hospital, Foshan 528000, China

**Keywords:** 腺癌, 鳞癌, 胸腔内播散, 随访观察, Adenocarcinoma, Squamous carcinoma, Thoracic dissemination, Observation

## Abstract

**背景与目的:**

手术非晚期期患者治疗的标准治疗，但是大量的回顾性研究显示胸腔内播散型肺癌接受主病灶切除后获益明显。非标准治疗之后患者该选择何种治疗策略？本研究通过回顾性数据去探究接受了主病灶切除的胸腔内播散型肺癌患者接下来何总治疗方式更优。

**方法:**

回顾性收集早期肺腺癌或肺鳞癌且复发模式为胸腔内播散型患者；或拟行肺癌根治术，但术中胸腔探查发现胸腔内播散，接受主病灶切除的肺腺癌或肺鳞癌患者的一般资料、病理、淋巴结状态、基因突变状态、初始治疗方式等，随访至进展、死亡或失访，记录患者无进展生存时间、总生存时间、从确诊到开始治疗的时间。通过*Kaplan*-*Meier*绘制生存曲线，*Log*-*rank*检验比较组间生存差异，*Cox*比例回归风险模型分析无进展生存期（progression-free survival, PFS）和总生存期（overall survival, OS）相关预后因子。

**结果:**

研究共纳入141例患者，70例r-M1a和71例s-M1a1患者。化疗组、靶向组、随访观察组患者中位PFS分别是14.7个月、41.0个月和31.0个月（95%CI: 19.01-26.01; *P* < 0.001），靶向治疗组和随访观察组患者PFS差异无统计学意义（*P*=0.600）。中位OS分别为39.0个月、42.6个月和38.1个月（95%CI: 32.47-45.33; *P*=0.478）。TTI < 3个月组和TTI≥3个月组患者的中位PFS分别是15.2个月和31.0个月（95%CI: 19.01-26.06; *P*<0.001），中位OS分别是41.7个月和38.7个月（95%CI: 32.47-45.33; *P*=0.714）。多因素分析显示性别（*P*=0.027）、淋巴结状态（*P*=0.036）、初始治疗方式（*P*<0.001）是PFS独立预后因子。

**结论:**

随访观察不会缩短胸腔内播散腺癌和鳞癌患者的生存时间，是一种可选的治疗策略。

## Abstract (en)

**Background and objective:**

Surgery was not standard-of-care of patients with advanced lung cancer. However, a serial of retrospective studies demonstrated that thoracic dissemination (M1a) patients could benefit from contraindicated surgery. After non-standard treatment, how should these patients choose following treatment approaches? Herein, we conducted this retrospective study to explore subsequent optimal treatment approaches.

**Methods:**

Different therapeutic approaches were evaluated by comparing progression-free survival (PFS), overall survival (OS), time to treatment interval (TTI) using the *Kaplan*-*Meier* method and *Log*-*rank* test. A *Cox* proportional hazards regression model was used for multivariate analysis.

**Results:**

141 eligible were enrolled. The median PFS of chemotherapy group, targeted therapy group and observation group were 14.7, 41.0 and 31.0 months, respectively (95%CI: 19.01-26.01; *P*<0.001). There was no significantly statistically difference between median PFS of targeted group and observation group (*P*=0.006). The median OS were 39.0, 42.6 and 38.1 months (95%CI: 32.47-45.33; *P*=0.478). The median PFS and OS of TTI < 3 months and TTI ≥3 months were 15.2 months versus 31.0 months (95%CI: 19.01-26.06; *P*<0.001) and 41.7 months versus 38.7 months (95%CI: 32.47-45.33; *P*=0.714). Multivariate analyses revealed gender (*P*=0.027), lymph node status (*P*=0.036) and initial therapy (*P*<0.001) were independent prognostic factors for PFS.

**Conclusion:**

Observation did not shorten survival of thoracic dissemination patients with lung adenocarcinoma or squamous carcinoma, therefore, it could be an favorable option. But prospective randomized controlled study was needed to confirm its validity.

肺癌是一种高度异质性^[1]^，并且预后相对较差的一种肿瘤性疾病^[2]^。远处器官的转移是是导致肺癌相关死亡的最主要原因^[3-5]^。呼吸系统转移在所有系统转移中居于第四位，约占所有肺癌转移的18%，占肺腺癌转移的22%^[6]^。非小细胞肺癌中胸膜转移的发生率在1%-7%，其中胸膜转移合并其他部位转移的发生率高达10%^[7, 8]^。晚期肺癌被认为是一种全身性疾病，治疗方式以系统治疗为主。当前晚期非小细胞肺癌标准一线治疗方式主要有化疗、靶向和免疫治疗，治疗方式的选择主要取决于基因状态和PD-L1表达或肿瘤突变负荷等。根据国际肺癌研究协会（International Association for the Study of Lung Cancer, IASLC）第八版TNM分期，胸腔内播散型肺癌（M1a）的中位生存时间只有11.5个月。手术被认为是Ⅳ期肺癌患者的禁忌证，但是一系列回顾性研究的结果显示接受了主病灶切除的胸腔内播散非小细胞肺癌患者的中位生存时间在13个月-64个月^[9-16]^，最短的中位生存时间也长于11.5个月。Xu等^[17]^的一项*meta*分析的结果同样也显示，胸腔内播散型肺癌患者能从主病灶切除的手术中获益。非适应症手术治疗没有缩短胸腔内播散型非小细胞肺癌患者的生存时间，反而延长了患者的生存。但是根据目前的指南^[18]^，手术仍为非标准治疗。那么接受了非标准手术治疗的胸腔内播散的非小细胞肺癌患者接下来该采用何种治疗方式？临床上治疗治疗因医生而异。鳞癌和腺癌是非小细胞肺癌最常见的病理类型，因此，本研究通过回顾性数据数据尝试去探索主病灶切除的胸腔内播散的腺癌和鳞癌患者接下来最佳的治疗方式。

## 1 资料与方法

回顾性连续搜集2009年-2012年在广东省人民医院接受肺癌根治术的Ⅰa期-Ⅲa期腺癌和鳞癌患者并且复发模式为胸腔内播散（r-M1a），排除合并其他部位转移患，排除5年内发生其他部位恶性肿瘤者，r-M1a组共纳入患者70例。连续性搜集2009年到2016年术前临床诊断为Ⅰa期-Ⅲa期可手术的腺癌和鳞癌患者，拟行肺癌根治术，但术中探查意外发现胸腔内播散（s-M1a），并且胸腔内播散经术中冰冻和术后石蜡病理证实，后患者接受主病灶切除。主病灶切除方式包括，限制性切除和扩大切除，限制性切除包括楔形切除和肺段切除，扩大切除包括肺叶切除和全肺切除。排除胸腔探查术患者，排除排除5年内发生其他部位恶性肿瘤患者，共纳入s-M1a患者71例。患者的治疗方式会在患者或家属充分知情同意的前提下，根据患者的基因状态、一般状态评分、肺功能状态等，由经验丰富的肺肿瘤专科医生推荐，患者及家属根据自身意愿选择相应的治疗方式。本研究仅纳入初始治疗方式为化疗、靶向和随访观察的患者，剔除仅接受局部治疗患者，其中局部治疗包括，胸腔内灌注化疗、消融、放疗、多次手术切除转移灶等。根据初始治疗方式的不同，所有患者可以分为化疗组、靶向组和随访观察组。患者在确诊M1a后3个月内未接受任何抗肿瘤治疗且疾病未进展的患者纳入到随访观察组，不管3个月后患者是否其他的抗肿瘤治疗；患者在确诊M1a后的3个月内一线治疗为化疗者纳入到化疗组；同样，一线治疗为靶向治疗者，纳入到靶向治疗组。

所有患者建议门诊规律随访。确诊前2年，建议每2个月-3个月复查一次胸部计算机断层扫描（computed tomography, CT）和血清癌胚抗原（carcinoembryonic antigen, CEA）；2年后每3个月-6个月复诊一次。但患者有任何肺癌相关症状应立即就诊，根据情况行脑CT或磁共振成像（magnetic resonance imaging, MRI）、正电子发射型计算机断层显像（positron emission tomography-computed tomography, PET-CT）、支气管镜、淋巴结活检等其他相关检查。

搜集患者的一般临床资料、病理、基因状态和治疗方式，包括性别、年龄、吸烟状态、一般状态评分（performance status, PS），淋巴结转移情况、表皮生长因子受体（epithelial growth factor receptor, EGFR）、间变性淋巴瘤激酶（anaplastic lymphoma kinase, ALK）重排情况、确诊时症状有无、初始治疗方式、手术方式等。通过*Pearson* *Chi*-*Square*检验、*Fisher*精确概率法比较不同治疗组间患者差异。总生存时间（overall survival, OS）定义为从诊断到因为任何原因导致的死亡或者失访的时间长度；无进展生存期（progression-free survival, PFS）为从诊断到第一次影像明确记录的进展、死亡或失访的时间段。无治疗间歇期（time to treatment interval, TTI）是指未在患者从确诊到接受抗肿瘤的时间期。通过*Kaplan*-*Meier*绘制生存曲线，*Log*-*rank*检验比较不同治疗方式组间生存差异，*Cox*比例风险回归模型进行多因素分析。所有的结果以风险比（hazard ratio, HR）和95%CI呈现。双侧检验且*P* < 0.05才被认为是有统计学意义。数据分析采用SPSS 22.0（IBM, Armonk, NY）软件进行。

## 2 结果

本研究共纳入141例患者，r-M1a组70例，s-M1a组71例，按照初始治疗方式可以分为化疗组、靶向组和随访观察组，纳入患者例数分别为57例、24例和60例。患者基线特征如表 1所示。其中男性79例（56.0%），女性62例（44.0%），平均年龄为56.7（23-81）岁。38例（27.0%）患者既往有吸烟史，其余均为非吸烟者。所有患者的一般状态评分均为0或1分。大部分（92.9%）为肺腺癌患者，仅有10例（7.1%）为鳞癌患者。53.2%的患者淋巴结状态为N0，其次分别为N2（31.9%）、N1（12.8%）和N3（2.1%）。*EGFR*突变患者共77例（54.6%），其中随访观察组有最高比例（22.7%）的*EGFR*突变患者。大部分患者的ALK重排为野生型，占比约64.5%，还有34.0%的患者无ALK检测数据。仅有20.6%的患者在确诊时存在肺癌相关的症状。化疗组、靶向组、随访观察组除年龄和组别外，其他患者基线特征差异均无统计学意义。

**1:** 患者基线特征比较 Comparison of baseline characteristics

Clinical features		Total (*n*=141)	Chemotherapy group (*n*=57)	Targeted group (*n*=24)	Follow-up observation group (*n*=60)	*P*
Gender	Male	79(56.0%)	34(24.1%)	15(10.6%)	30(21.3%)	0.450
	Female	62(44.0%)	23(16.3%)	9(6.4%)	30(21.3%)	
Age(yr)	≤65	105(74.5%)	48(34.0%)	19(13.5%)	38(27.0%)	0.030
	> 65	36(25.5%)	9(6.4%)	5(3.5%)	22(15.6%)	
PS	0-1	141(100.0%)	57(40.4%)	24(17.0%)	60(42.6%)	
Smoking	No	103(73.0%)	36(25.5%)	18(12.8%)	49(34.8%)	0.076
histroy	Yas	38(27.0%)	21(14.9%)	6(4.3%)	11(7.8%)	
Pathological	Adenocarcinoma	131(92.9%)	50(35.5%)	24(17.0%)	57(40.4%)	0.102
type	Squamous cell arcinoma	10(7.1%)	7(5.0%)	0(0.0%)	3(2.1%)	
Lymph node	N0	75(53.2%)	29(20.6%)	13(9.2%)	33(23.4%)	0.436
status	N1	18(12.8%)	9(6.4%)	2(1.4%)	7(5.0%)	
	N2	45(31.9%)	16(11.3%)	9(6.4%)	20(14.2%)	
	N3	3(2.1%)	3(2.1%)	0(0.0%)	0(0.0%)	
EGFR	Mutant	77(54.6%)	26(18.4%)	19(13.5%)	32(22.7%)	0.069
	Wild type	46(32.6%)	24(17.0%)	3(2.1%)	19(13.5%)	
	Unknown	18(12.8%)	7(5.0%)	2(1.4%)	9(6.4%)	
ALK	Rearrangement	2(1.4%)	2(1.4%)	0(0.0%)	0(0.0%)	0.368
	Wild type	91(64.5%)	34(24.1%)	18(12.8%)	39(27.7%)	
	Unknown	48(34.0%)	21(14.9%)	6(4.3%)	21(14.9%)	
Group	r-M1a s-M1a	70(49.6%) 71(50.4%)	21(14.9%) 36(25.5%)	10(7.1%) 14(9.9%)	39(27.7%) 21(14.9%)	0.007
Symptom	No	48(34.0%)	20(14.2%)	9(6.4%)	19(13.5%)	0.164
	Yes	29(20.6%)	17(12.1%)	3(2.1%)	9(6.4%)	
	Unknown	64(45.4%)	20(14.2%)	12(8.5%)	32(22.7%)	

所有患者平均随访时长44.4（1.4-130.8）个月。r-M1a和s-M1a同属胸腔内播散型肺癌，生存分析显示r-M1a和s-M1a组患者的中位PFS和OS分别是27.5个月*vs* 21.9个月（*P*=0.134）和32.6个月*vs* 45.7个月（*P*=0.154），差异均无统计学意义。化疗组、靶向组、随访观察组患者中位PFS分别是14.7个月、41.0个月和31.0个月（95%CI: 19.01-26.01;*P*<0.001），进一步分析显示靶向治疗组和随访观察组患者PFS差异无统计学意义（*P*=0.600）（图 1A）。三组的中位OS分别为39.0个月、42.6个月和38.1个月（95%CI: 32.47-45.33; *P*=0.478）（图 1B）。患者的无治疗间歇期如图 2所示。我们统计发现在确诊后愿意接受抗肿瘤治疗的患者会在2.8个月内接受治疗。因此我们设定一个cut-off值为3个月，将所有患者分成TTI < 3个月组和TTI≥3个月。分析显示TTI < 3个月组和TTI≥3个月组患者的中位PFS分别是15.2个月和31.0个月（95%CI: 19.01-26.06;*P*<0.001）（图 3A），中位OS分别是41.7个月和38.7个月（95%CI: 32.47-45.33; *P*=0.714）（图 3B）。在TTI≥3个月组中，疾病进展前接受一线化疗、靶向、局部治疗和继续随访观察的患者分别有10例、14例、1例和35例，剔除局部治疗组1例，其中位PFS分别为22.9个月、22.2个月和35.5个月（95%CI: 14.50-49.4; *P*=0.153），中位OS分别为45.2个月、NR（未达到）和26.4个月（95%CI: 26.98-50.82; *P*=0.086），化疗组、靶向治疗组和随访观察组患者中位OS差异无统计学意义，但是趋势明显，化疗组和靶向治疗组预后好明显好于继续随访观察组（图 4）。*Cox*多因素分析显示性别（*P*=0.027）、淋巴结状态（*P*=0.036）、初始治疗方式（*P*<0.001）是PFS独立预后因子。男性患者的进展风险是女性患者的1.63倍；淋巴结转移患者有更高的进展风险，并且淋巴结分级越高进展风险越大；化疗组患者的进展风险是随访观察组患者的3.2倍。

**1:**
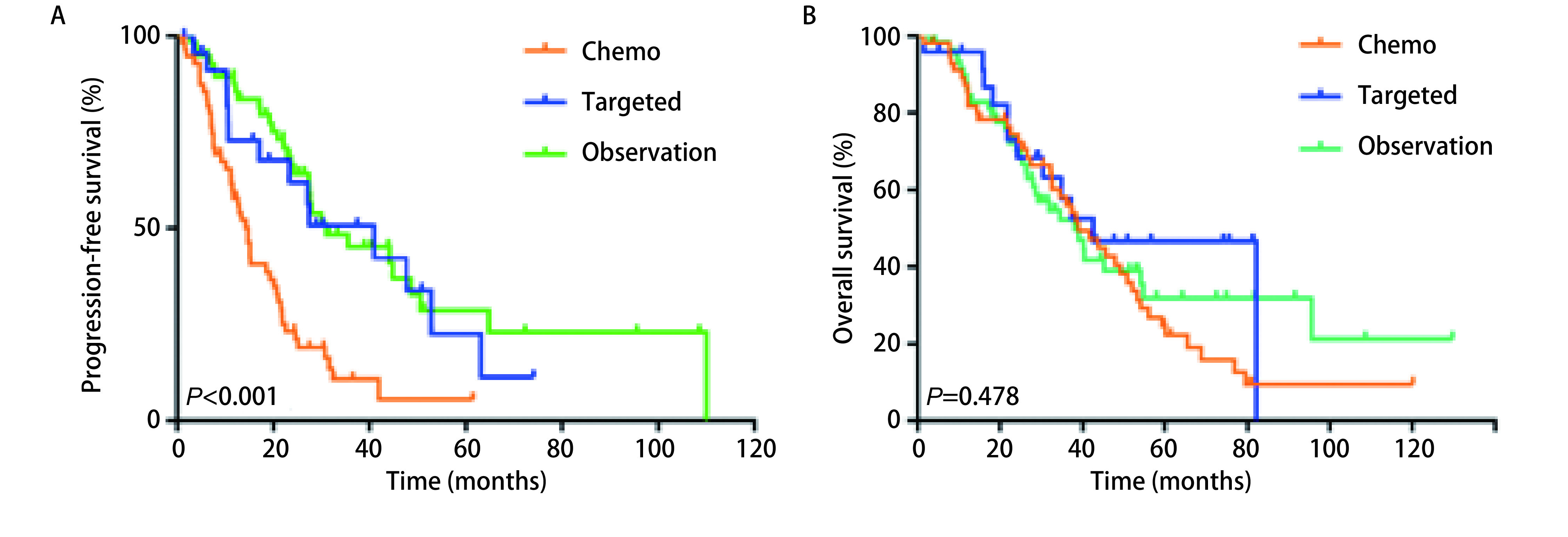
胸腔内播散肺腺癌和鳞癌患者初始治疗方式对患者生存的影响 The effect of initial treatment on the survival of patients with disseminated lung adenocarcinoma and squamous cell carcinoma in chest cavity

**2:**
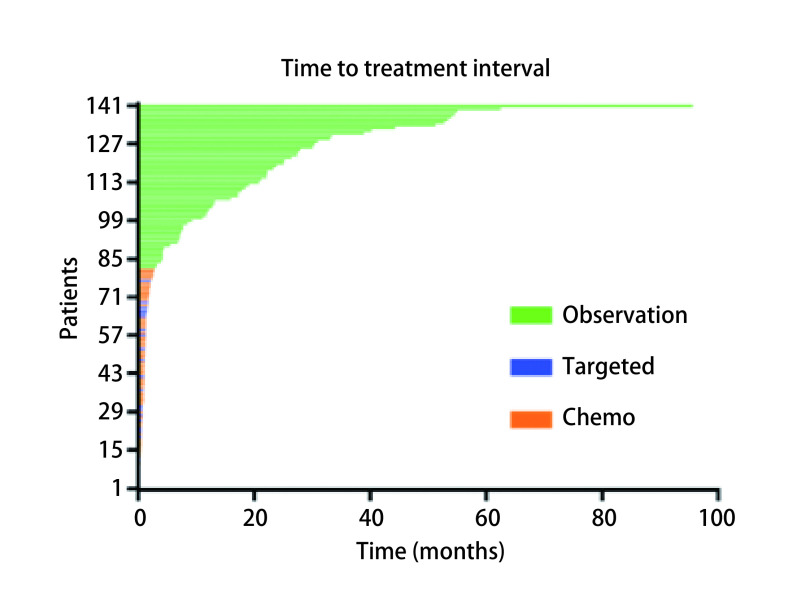
所有胸腔内播散肺腺癌和鳞癌患者无治疗间歇期 Time to treatment interval of all patients with disseminated lung adenocarcinoma and squamous cell carcinoma in the thoracic cavity

**3:**
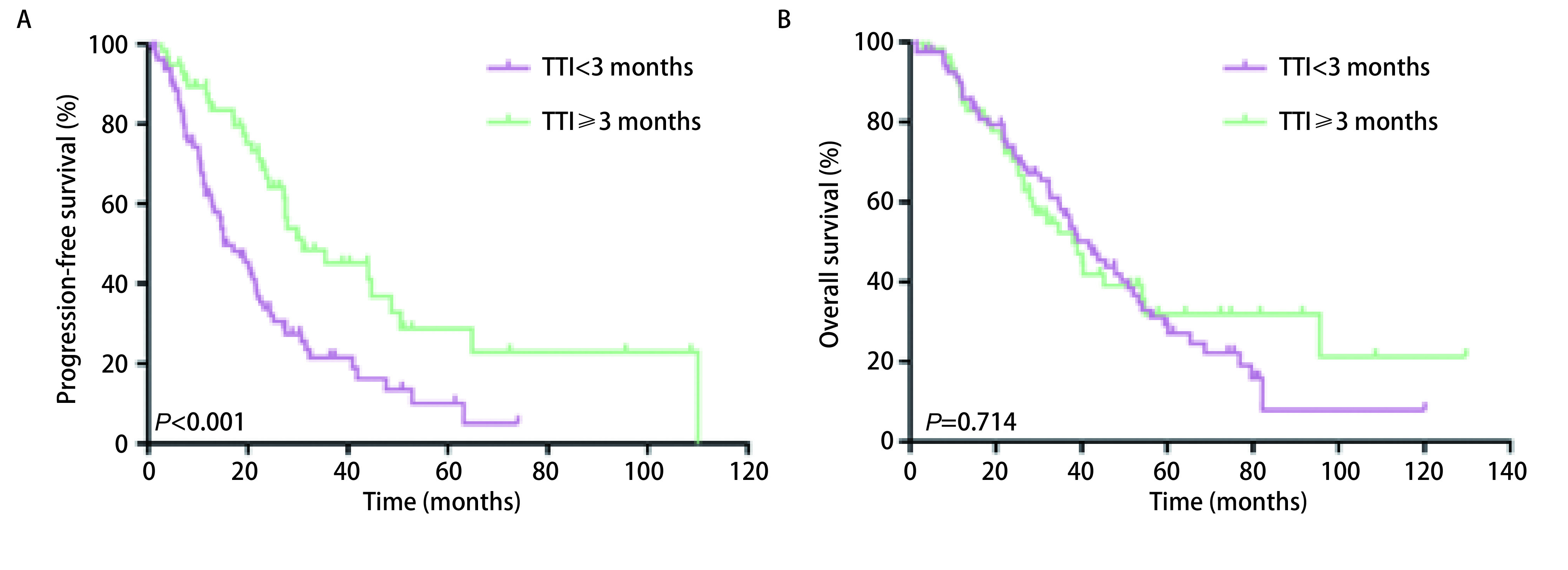
无治疗间歇期TTI < 3个月组和TTI≥3个月组患者生存比较 Comparison of survival between patients without TTI < 3 months in treatment interval and TTI ≥ 3 months

**4:**
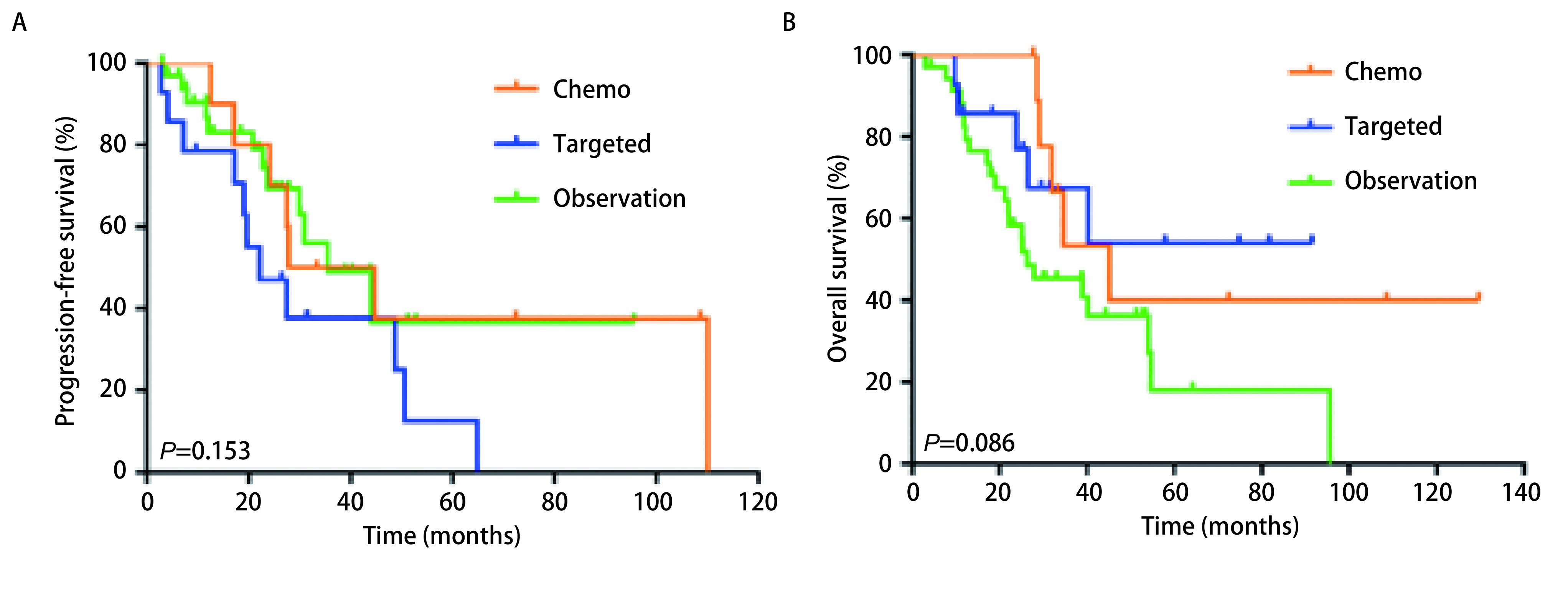
TTI≥3个月组患者一线治疗方式对患者生存的影响 Effect of first-line treatment on survival of patients with TTI≥3 months

## 3 讨论

晚期非小细胞肺癌被认为是一种全身性疾病，治疗方式以系统治疗为主。上个世纪末抗肿瘤治疗仍以化疗为主，直到1988年和2007年EGFR和ALK靶点才相继在肺癌治疗领域被发现^[19, 20]^。靶向药物更优的药物疗效更生活质量改善方面明显优于化疗，这使得传统的铂类为基础的双药细胞毒药物化疗逐渐转向针对特定靶点的靶向治疗。但是，对于无驱动基因突变的患者，化疗仍是其首选治疗方式。该研究所纳入的患者的一线治疗方式均为三代铂类为基础的双药化疗，一代EGFR酪氨酸激酶抑制剂（EGFR-TKI）——厄洛替尼（erlotinib）, 吉非替尼（gefitinib）和埃克替尼（icotinib）或ALK酪氨酸激酶抑制剂（ALK-TKI）——克唑替尼（crizotinib）。Martin F. Dietrich教授的一篇综述显示接受一线铂类为基础的双药化疗的患者PFS和OS分别为2.8个月-6.7个月和7.4个月-13.9个月^[21]^。*EGFR*敏感突变患者一线治疗为EGFR-TKI，患者的中位PFS和OS大致波动在8.0个月-13.1个月和19.3个月-35.5个月^[22-29]^。PROFILE 1014^[30]^、PROFILE 1029^[31]^和J-ALEX^[32]^研究结果显示ALK重排患者一线克唑替尼治疗，患者的中位PFS约为10.2个月-11.1个月。根据第8版世界肺癌协会TNM的分期的数据显示M1a患者的中位生存时间为11.5个月^[2]^。大量的回顾性数据显示接受主病灶切除的胸腔内播散非小细胞肺癌中位生存时间在13个月-64个月^[9-16]^。但是非标准手术治疗后，患者该采用何种治疗方式达到获益最大化，目前尚无这方面的研究。

该研究的M1a的鳞癌和腺癌患者的中位PFS和OS分别为22.5个月和38.9个月，显著长于11.5个月。这个既往的研究结果是想似的，非适应症手术并没有缩短M1a患者的生存时间。靶向和随访观察组患者的的PFS显著长于化疗组的14.7个月，靶向组和随访观察组的PFS虽然存在数据上10个月的差异，但是差异没有统计学意义。三组的中位OS差异无统计学意义。这说明初始治疗采用随访观察的策略也是可行的，并不会缩短患者的生存时间。进一步的分析显示，TTI≥3个月组患者的中位PFS是TTI < 3个月组的2倍，但是OS无差异。TTI的存在一定程度上延长了患者的无进展生存时间，并且没有影响患者的生存时间。多因素分析显示女性患者的预后相对更好；淋巴结转移患者有更高的进展风险，并且淋巴结分级越高进展风险越大。化疗组患者的进展风险是随访观察组患者的3.2倍。因此对于无主病灶的M1a的腺癌和鳞癌患者，尤其是女性，淋巴结分期为N0的患者，密切随访的过程中采用观察的策略也是可选的。在TTI≥3个月组中疾病进展前有41.7%的患者接受了抗肿瘤治疗。患者的PFS和OS均无统计学差异，但是存在一种趋势，即继续随访观察组患者的OS是明显短于化疗和靶向治疗组的。由于大部分患者无靶病灶，因此我们纳入的患者的干预时机是当患者患者肿瘤相关症状或肺部病灶出现缓慢增大趋势时及时进行干预。

综上所述，随访观察不会缩短胸腔内播散的肺腺癌和鳞癌患者的生存时间，甚至在一定程度上可以延长这类患者的无进展生存，因此随访观察胸腔内播散的肺腺癌和鳞癌患者是一种可选的治疗策略。
